# A Review of Breast Cancer Risk Factors in Adolescents and Young Adults

**DOI:** 10.3390/cancers13215552

**Published:** 2021-11-05

**Authors:** Una Mary McVeigh, John William Tepper, Terri Patricia McVeigh

**Affiliations:** 1 School of Medicine, National University of Ireland Galway, H91 TK33 Galway, Ireland; u.mcveigh1@nuigalway.ie; 2 Department of Internal Medicine/Pediatrics, Indiana University School of Medicine, Indianapolis, IN 46202-3082, USA; jwtepper@iu.edu; 3 Royal Marsden NHS Foundation Trust, London SW3 6JJ, UK; 4 Institute of Cancer Research, London SW7 3RP, UK

**Keywords:** breast cancer, AYA cancer, risk prediction, inherited risk

## Abstract

**Simple Summary:**

Cancer diagnosed in patients between the ages of 15 and 39 deserves special consideration. Diagnoses within this cohort of adolescents and young adults include childhood cancers which present at an older age than expected, or an early presentation of cancers that are typically observed in older adults, such as breast cancer. Cancers within this age group are associated with worse disease-free and overall survival rates, and the incidence of these cases are rising. Knowing an individual’s susceptibility to disease can change their clinical management and allow for the risk-testing of relatives. This review discusses the risk factors that contribute to breast cancer in this unique cohort of patients, including inherited genetic risk factors, as well as environmental and lifestyle factors. We also describe risk models that allow clinicians to quantify a patient’s lifetime risk of developing disease.

**Abstract:**

Cancer in adolescents and young adults (AYAs) deserves special consideration for several reasons. AYA cancers encompass paediatric malignancies that present at an older age than expected, or early-onset of cancers that are typically observed in adults. However, disease diagnosed in the AYA population is distinct to those same cancers which are diagnosed in a paediatric or older adult setting. Worse disease-free and overall survival outcomes are observed in the AYA setting, and the incidence of AYA cancers is increasing. Knowledge of an individual’s underlying cancer predisposition can influence their clinical care and may facilitate early tumour surveillance strategies and cascade testing of at-risk relatives. This information can further influence reproductive decision making. In this review we discuss the risk factors contributing to AYA breast cancer, such as heritable predisposition, environmental, and lifestyle factors. We also describe a number of risk models which incorporate genetic factors that aid clinicians in quantifying an individual’s lifetime risk of disease.

## 1. Cancer in Adolescents and Young Adults

Adolescents and young adults (AYAs) with cancer, who are defined as those individuals diagnosed with cancer between the ages of 15 and 39 [1], deserve special consideration for a number of reasons. Cancers in this cohort may include those more typically identified in older adults that occur at unexpectedly young ages, or, conversely, more typically paediatric cancers that are diagnosed later in life. The most common types of cancers diagnosed in this population include breast, cervical, and thyroid cancers, along with melanoma, haematological malignancies, and central nervous system tumours [2,3]. The incidence of cancer diagnoses in AYAs has increased significantly since 2000 [4]. There are several indicators that the diseases that are diagnosed in the AYA population is distinct to those same cancers diagnosed in a paediatric or older adult setting. Disease-free and overall survival outcomes in the AYA setting are worse, and differences in the biology and epidemiology of these tumours have been observed [1,5,6,7]. There is evidence that AYA patients present with a more aggressive disease and use mastectomy and chemotherapy at higher rates than adult breast cancer patients [8,9,10,11,12]. Furthermore, AYAs have additional complicating factors related to fertility preservation and family planning, as well as psychosocial implications. There is an associated financial impact, with an associated loss of earnings related to time off work, as well as treatment-related costs.

Underlying hereditary cancer susceptibility syndromes account for 7–10% of cancer diagnoses in children and adolescents [13,14,15,16]. For young adults, moderate- to high-risk cancer susceptibility variants have been associated with 5.9–23% of early-onset cancers, including invasive breast cancer [17,18,19,20,21,22,23]. Knowledge of an affected patient’s underlying high-risk genotype can influence clinical care in terms of surgical and chemotherapeutic decision-making, as well as their future cancer risk estimation, while in the unaffected individual, early intervention with regular screening, as well as chemo- and/or surgical prophylaxis may minimise cancer risk, or at least facilitate early diagnosis and treatment. In AYAs, identifying a monogenic risk factor for cancer may also influence reproductive decision making, and may facilitate options such as pre-implantation genetic diagnosis, or non-invasive/invasive prenatal testing.

## 2. Epidemiology and Aetiology of Breast Cancer

Breast cancer among cis-gender females is very common, affecting approximately 1 in 8 women over their lifetime, with the peak age-specific incidence between the ages of 65 and 69 (417.3 per 100,000), when compared to an incidence of 109.2 per 100,000 in those aged under 40 [2]. Female breast cancer diagnosis before age 30 is particularly rare (incidence rate 12.2/100,000). Male (cis-male) breast cancer is rare at any age, with an overall incidence of 1.3 per 100,000; however, cases before age 40 are particularly infrequent [2].

The risk of breast cancer is dependent on several so-called modifiable factors, which include body weight, use of hormone replacement therapy, and reproductive factors, as well as non-modifiable factors such as age at menarche and menopause, breast density, and a family history of breast cancer. Twin studies demonstrated that 30% of breast cancers are attributed to hereditary factors [24]. Breast cancer incidence is twice as high in women with at least one affected first-degree relative [25]. There is an observable Mendelian inheritance pattern in 5–10% of all breast cancers, in which case the cancer risk is considered a hereditary trait [26]. A further 20% of all breast cancers are considered “familial”, where familial clustering of affected first- or second-degree relatives is observed in the absence of an obvious monogenic variant [27].

Research over the past three decades has identified germline variants at numerous loci with variable associated disease penetrance. The genetic variants that predispose a person to breast cancer are now broadly categorised into three groups: high-, moderate-, and low-risk variants, depending on the relative risk conferred by the variant. Pathogenic high-risk variants in several genes are associated with a greater than four-fold risk of breast cancer than that observed in the general population [28], and moderate-risk in the order of 2–4 times greater than population risk [29,30,31,32].

In keeping with the rules governing biological fitness, it follows that those variants that are associated with a particularly high risk of early-onset disease are usually rare in the general population when compared to low-risk susceptibility alleles, which may be relatively common [33]. Risk alleles are not mutually exclusive, and other environmental or lifestyle risk factors can modify the absolute risk of disease associated with a genetic trait, such that a carrier of a so-called “moderate-risk” variant allele may have an overall high risk, when a holistic risk assessment is undertaken.

## 3. Monogenic Predisposition to Breast Cancer

Individuals who carry inherited genetic traits that are associated with a high risk of cancer can manage their cancer risk through a combination of surveillance, risk-reducing surgery or other techniques. The exact advice provided to carriers of such traits will depend on the genotype, associated cancer risk and the availability of proven screening or other risk-reducing interventions. The early recognition of a genetic predisposition to disease, as well as the potential barriers to genetic testing where such a predisposition is suspected, is crucial to facilitate cascade testing and early intervention in at-risk relatives [34].

### 3.1. TP53

*TP53* encodes for the p53 protein, which is commonly referred to as the “guardian of the genome” [35]. In response to cellular stress, DNA damage, and oncogene activation, p53 regulates the expression of thousands of other genes to induce DNA repair, cell cycle arrest, apoptosis, or senescence. Pathogenic variants in *TP53* may cause conformational changes in the protein, abrogate DNA binding or suppress interaction with target genes. Deleterious variants may cause a partial or total loss of key functions, and certain deleterious variants may lead to an inappropriate gain of function—such as inappropriate transcriptional activity [35]. Germline variants in certain tumour suppressor genes follow the two-hit hypothesis [36], behaving recessively at the cellular level, such that loss of function is not apparent until a second somatic hit (deletion, variation or silencing by methylation) occurs in the normal allele—leading to a demonstrable loss of heterozygosity. In contrast, this may not necessarily be evident in the case of *TP53*—certain *TP53* variants may exert dominant negative effects on the wild type allele, for example, by inactivation of the wild-type protein or through dimerization [37].

Disruption of p53 function due to germline or somatic pathogenic variants contributes to virtually all of the hallmark features of cancer [35]. Somatic *TP53* variants are common across a host of cancer types, most commonly colorectal and head and neck cancer, and TP53-mutant cancers demonstrate higher rates of chemo- and or radio-therapeutic resistance, increased risk of relapse, and reduced survival [38,39].

Heterozygous pathogenic germline variants of *TP53* give rise to heritable TP53-related cancer (hTP53rc) syndrome, of which the most extreme phenotype is traditionally referred to as Li Fraumeni syndrome (LFS) [40]. This rare syndrome predisposes affected individuals to a wide range of malignancies, including sarcoma of the soft tissue and bone, brain tumours, haematological malignancies, adrenocortical cancer, and breast cancer, among others, with this cancer risk starting from early childhood [40].

Most pathogenic *TP53* variants are associated with a higher penetrance than *BRCA* variants [41,42,43], although variants with a reduced penetrance have been reported. The median age of a diagnosis of breast cancer, in a female carrier of a germline pathogenic variant in *TP53*, is 33 years of age, with approximately one third of this population diagnosed prior to the age of 30 years. Indeed, overall, approximately 50% of the cancers occurring in the carriers of such variants occur before this age. The population frequency of germline pathogenic variants in *TP53* is very low, ranging from 1 in 3555 to 1 in 5476 individuals [44], although the prevalence is higher in certain populations where founder events have been reported [45]. In women diagnosed with breast cancer before age 30, the likelihood of detecting an underlying germline *TP53* variant is 2–8% [46]. The likelihood of detecting such variants in women with breast cancer decreases with age, although certain variants have been reported to be associated with a later age of diagnosis [47]. The likelihood of identifying an underlying germline *TP53* variant is also dependent on the molecular phenotype of the tumour. There is an apparent enrichment for HER2-overexpression, and particularly “triple positive” (ER+, PR+ and HER2+) breast cancers —compared to the general population, in which HER2-overexpressing breast cancers account for 20–30% of cases, 63–83% of breast cancers in carriers of pathogenic *TP53* variants are HER2-amplified [48,49]. Interestingly, the risk of male breast cancer does not appear to be significantly increased [40]. Malignant Phyllodes tumours have also been reported in this population [41].

Given the risk of early-onset cancer, it is advised that carriers of pathogenic *TP53* variants have an annual clinical screening and a whole-body MRI (WBMRI), with an annual breast MRI beginning at 20 years of age [40]. A discussion regarding prophylactic risk reducing surgery may be appropriate [50].

### 3.2. BRCA1 and BRCA2

Since their discovery over three decades ago, the breast cancer 1 (*BRCA1*) and 2 (*BRCA2*) genes remain the most clinically significant breast cancer predisposition genes. A multi-functional protein, BRCA1 is involved in a wide array of cellular pathways, including DNA damage repair (DDR), chromatin remodelling, gene expression, and protein ubiquitination [51,52,53,54]. BRCA2 is also involved in DDR, primarily as part of the homologous repair pathway (HRR) [55,56].

Heterozygous carriers of germline pathogenic variants in either *BRCA1* or *BRCA2* are at significantly increased lifetime risk of breast and other cancers, with risk accruing from a young age. It is estimated that between 1 in 300 and 1 in 200 individuals in the general population carry a germline pathogenic variant in one of these genes [57,58,59]. There are recurrent founder events in these genes; approximately 1 in 40 individuals of Ashkenazi Jewish heritage will carry a germline pathogenic variant in either *BRCA1* or *BRCA2*, and most of these identified variants will be one of three founder mutations [60]. In women with breast cancer, the prevalence of pathogenic *BRCA1*/*BRCA2* variants is approximately 1 in 33 [58], and almost 1 in 8 if the affected individual is an AYA [17,58,61].

The cumulative lifetime risk of breast cancer that is conferred by pathogenic *BRCA1* and *BRCA2* variants is estimated to be 65–79% and 61–77%, respectively [62]. There is evidence to suggest that the cancers occurring in the carriers of germline *BRCA* variants result in more aggressive malignancies at earlier ages. The cancer risk associated with pathogenic *BRCA1* variants peaks between 30 and 50 years [63], with a standardised incidence rate of 46.2 (37.3–57.1)% between the ages of 31 and 40 years compared to 7 (4.5–11)% in those between 61 and 70 years [62]. The majority of breast cancers in patients who carry a germline *BRCA1* variant are the basal subtype of triple negative breast cancer (TNBC) [64,65,66]. There is some evidence that *BRCA2* variants show more association with the Luminal B subtype, and a higher risk of contralateral disease, but no definitive association has been demonstrated thus far [66,67,68]. Germline *BRCA* variants are associated with more aggressive malignancies at earlier ages, presenting with a higher grade and metastatic potential. Tumours that are associated with a germline variant are also associated with a poorer prognosis than those that acquire a somatic *BRCA* variant [69].

The risk of male breast cancer is also increased in the male carriers of such variants, particularly those in *BRCA2*, where the lifetime risk is approximately 8–10% when compared to *BRCA1*, where the risk is in the order of 1% [70]. The risk of prostate cancer is also particularly increased in the male carriers of *BRCA2* variants, with a lifetime risk of approximately 25–30%, and a tendency to a more aggressive phenotype. The risk of prostate cancer in male carriers of *BRCA1* variants is approximately equivalent to the general population’s risk, but the phenotype of such cancers, as well as the therapeutic approach, is similar to that of carriers of *BRCA2* variants. Female carriers of BRCA1/BRCA2 variants also have the additional high risk of ovarian cancer, with the cumulative lifetime risk in carriers of *BRCA1* being 44% (36–53%), compared to *BRCA2*, which is 17% (11–25%) [62]. Both female and male carriers have increased risks of pancreatic cancer [71], and there is also an increased risk of melanoma-type skin cancer in carriers of *BRCA2* variants [72].

In unaffected women found to carry pathogenic/likely pathogenic variants in *BRCA1* or *BRCA2*, high intensity surveillance with MRIs and/or mammograms (depending on age and breast density) is recommended, starting from 25–30 years depending on family history and local guidelines [73,74]. The carriers of such variants may wish to consider surgical prophylaxis, although the survival benefit of this when compared to regular surveillance is uncertain; any potential benefit diminishes rapidly with increasing age at surgical intervention [75,76,77]. Carriers of *BRCA* variants who have already developed breast cancer are also predisposed to developing further primary breast cancers in any residual breast tissue. Consequently, affected patients may choose to have contralateral prophylactic surgery during or after therapeutic surgery, although several factors influence the potential survival benefit of this approach, including age and the stage of the first breast cancer, systemic treatments, age at prophylactic surgery, and surveillance [78]. The benefit of risk-reducing mastectomy after a diagnosis of ovarian cancer is uncertain, and the life expectancy of a patient with *BRCA*-associated ovarian cancer may be too short to warrant preventive surgery. However, such surgery may be considered in individuals with occult, or stage I/II ovarian cancer, which is diagnosed before age 55, with at least 10 years of disease-free survival [79].

As well as contributing significantly to the risk of disease, *BRCA*-deficiency modulates the response of disease to systemic cancer treatment. Loss of BRCA1 or BRCA2 function, as a consequence of biallelic variation or hypermethylation (germline and/or somatic), leads to homologous recombination repair deficiency, which can then be exploited for therapeutic purposes. Such a deficiency is characterised by a unique mutational signature, and a high genomic instability score (GIS), which is derived from an algorithmic measurement of large-scale state transitions, telomeric allelic imbalance, and loss of heterozygosity [80]. Tumours in *BRCA* variant carriers demonstrate a higher objective response rate to platinum-based chemotherapy (carboplatin) versus taxane chemotherapy (docetaxel) (68% vs. 33%) [81]. High rates of pathological complete remission have also been demonstrated in *BRCA1* variant carriers who are treated with platinum-based neoadjuvant chemotherapy [82]. These data highlight the importance of *BRCA* variant screening in patients with TNBC. However, it has been reported that secondary tumours develop resistance to platinum-based therapies via a somatic reversion mutation which restores BRCA function [83]. This same mechanism could result in resistance to another therapeutic option: PARP-inhibitors.

The use of PARPi in BRCA-deficient tumours has been shown to be particularly effective [84,85,86]. In the carriers of germline pathogenic variants in *BRCA1* or *BRCA2*, olaparib treatment has been shown to lead to superior progression-free survival when compared to standard therapy in individuals with HER2-negative metastatic breast cancer [87], and to longer disease-free survival in individuals with high-risk HER2-negative early breast cancer when compared to placebo [88]. The efficacy of PARP inhibitors in the therapeutic context has prompted much interest in their potential application in chemoprophylaxis. PARP inhibitors have been shown to delay mammary tumour development in BRCA-deficient murine models [89,90]. Further research is required to determine their efficacy in the delay or prevention of cancer onset in the carriers of germline *BRCA1*/*BRCA2* variants, as well as to investigate their potential toxicity, risk of secondary cancer development and any mechanisms of resistance [91,92]. In the UK, for chemoprevention in unaffected women with a moderate (17–30%) or high (>30%) risk of breast cancer, the National Institute for Health and Care Excellence has recommended the consideration of tamoxifen, raloxifene or anastrozole, depending on menopausal status, the presence of a uterus, and history of contra-indications such as osteoporosis, endometrial cancer or thrombo-embolic disease [74]. Given their roles as selective oestrogen receptor modulators and aromatase inhibitors, the impact on breast cancer risk reduction is largely limited to oestrogen receptor-positive cancers [93]. Their utility in the carriers of pathogenic *BRCA1* variants is therefore limited, considering their strong association with ER-negative disease [94]. Other agents, including denosumab and metformin have also been investigated as chemoprophylactics [95].

Ovarian cancer screening in individuals with germline variants in *BRCA1* or *BRCA2* is contentious. Annual screening with CA-125 and transvaginal ultrasound has a high sensitivity and positive predictive value, but the majority of cancers detected in this manner are stage III or higher, and an annual screening is interval associated with a poor overall 10-year survival in the carriers of germline *BRCA1*/*BRCA2* variants [96]. The United Kingdom Collaborative Trial of Ovarian Cancer Screening (UKCTOCS) investigated the role of multimodal screening, with the use of serial CA-125 measurements (in the context of Risk of Ovarian Cancer Algorithm, ROCA), and second-line transvaginal ultrasound (TVUS) [97]. This study demonstrated a stage shift but did not provide conclusive evidence of survival benefit. At present, the only proven way to minimise the risk of ovarian cancer in the carriers of pathogenic variants in *BRCA1*/*BRCA2* is prophylactic bilateral salpingo-oophorectomy. The optimum age at which this is undertaken should consider age-related penetrance, and the adverse impact of premature menopause on heart, brain and bone health. Risk-reducing salpingo-oophorectomy is associated with a 96% reduction in the risk of tubo-ovarian cancer; and may be associated with a reduction in breast cancer risk in the carriers of *BRCA2*, but not *BRCA1*, variants [98,99].

### 3.3. PALB2

The tumour suppressor PALB2 mediates the physical interaction of BRCA2 with the COOH-terminal fragment of BRCA1 [100]. Heterozygous pathogenic germline variants in *PALB2* have been associated with an increased risk of breast cancer which approaches that seen with variants in *BRCA2*, in the order of 48–63%, with increased risks for those patients with a family history of breast cancer [101,102,103,104,105]. The risks associated with germline pathogenic variants in *PALB2* are highest for those under 40 years of age, at 8–9 times the risk of the general population, and while this falls somewhat with age, this risk remains significant, with a 5-fold relative risk in those carriers aged over 60 years [102,105]. Male breast cancers have been reported in the carriers of *PALB2* variants, but the absolute risk appears very low 0.9 (0.2–5)%. Additionally, female carriers of *PALB2* variants have increased risks of ovarian cancer in the order of 5 (2–10)%, and carriers of either gender have an increased risk of pancreatic cancer (approximately 2–3%).

BRCA-equivalent breast surveillance is recommended for the carriers of pathogenic variants in *PALB2*. Depending on the specific variant identified and the patient’s family history, prophylactic risk-reducing breast surgery may be considered, although specific data regarding a survival advantage in carriers of *PALB2* variants is lacking [50,106]. At present, there is insufficient evidence to recommend risk-reducing ovarian surgery in the carriers of PALB2 variants, but, in the absence of a proven benefit of ovarian cancer screening, it could be considered in the post-menopausal setting, after non-directive counselling.

PALB2 has a critical role in homologous recombination repair, such that tumours which are associated with pathogenic variants in PALB2 demonstrate homologous recombination repair deficiency [107], and there is increasing evidence to suggest that such tumours may demonstrate a favourable response to platinum-based chemotherapy and PARP inhibitors, such that the American College of Medical Genetics recommends the consideration of the same systemic options for the carriers of *PALB2* variants as for carriers of pathogenic variants in *BRCA1*/*BRCA2* [106].

### 3.4. RAD51C and RAD51D

Two other proteins that function in the homology-directed repair of double stranded DNA breaks are RAD51C and RAD51D, which are encoded for by the *RAD51C* and *RAD51D* genes, respectively. The association between germline pathogenic variants in these genes and ovarian cancer has been recognised for many years, but the association with breast cancer risk was only more recently established [108,109]. Previous studies have shown a particularly strong association between pathogenic *RAD51C* and *RAD51D* variants and oestrogen receptor negative or triple-negative breast cancer [110,111]. The breast cancer risk associated with such variants appears to be moderate, and is significantly modified by age and family history, with relative risks higher in those aged 20–49, and in those with a family history of early-onset breast cancer [109].

### 3.5. Fanconi Anaemia

Fanconi anaemia (FA) is a rare, genetically heterogeneous disorder that is associated with congenital anomalies, skeletal, ophthalmic, and genitourinary malformations, cutaneous manifestations, and a predisposition to malignancies—most typically haematological malignancies and squamous cell cancers. Fanconi anaemia can be inherited in an autosomal recessive manner, most commonly due to biallelic, pathogenic variants in *FANCA*, and less frequently due to biallelic variants in any of at least twenty genes, which include *BRCA2*, *PALB2*, *RAD51C*, and very rarely, *BRCA1* [112]. Biallelic variants in *BRCA1* were traditionally believed to either be embryonically lethal, or a rare cause of Fanconi anaemia; however, increasing numbers of cases are being reported with phenotypes which overlap between Fanconi anaemia and other chromosome instability syndromes, particularly Nijmegen Breakage Syndrome; this has an associated risk of early-onset breast and/or ovarian cancer predisposition [113,114,115,116]. The individuals affected by early-onset breast cancer in these reports had other suggestive syndromic features.

In a proband with suspicious clinical findings, a diagnosis of FA can be determined by chromosome breakage analysis of lymphocytes treated with diepoxybutane (DEB) and mitomycin C [117], with subsequent testing of germline DNA using a panel of known FA-associated genes to establish the underlying genotype [118].

A diagnosis of Fanconi anaemia in a family has wide-ranging implications—each sibling of an individual with autosomal recessive FA has a 25% risk of being affected with the same disorder, and a 50% risk of being a heterozygous carrier of one variant allele, while the parents of such individuals are likely to be heterozygous carriers. Heterozygous carriers of variants in a gene associated with autosomal recessive forms of FA are not at risk of FA, but may be at increased risk of breast, ovarian, and/or other cancers, if the monoallelic variants in question are in *BRCA1*, *BRCA2*, *PALB2*, *BRIP1* or *RAD51C*. However, some FA-associated variants may be hypo-morphic, and cause FA only when found in trans with a different pathogenic variant. Heterozygous carriers of hypo-morphic alleles in cancer predisposition genes may be at an only moderately, or slightly, increased cancer risk [119].

### 3.6. ATM

ATM plays a major role in maintaining genomic stability where it functions in the DNA damage response, but also in controlling the cell cycle and mitotic recombination [120,121]. Biallelic pathogenic variants in *ATM* give rise to ataxia-telangiectasia syndrome. The features of ataxia-telangiectasia syndrome are usually evident from childhood, and include progressive ataxia, cutaneous and ophthalmic telangiectasia, as well as an increased risk of cancer. The most common malignancies in individuals with A-T are haematological, including leukaemias and lymphomas, however, solid organ cancers, including breast cancers, are being increasingly noted as the survival of affected individuals improves, as well as in the carriers of certain ATM haplotypes or hypo-morphic alleles. The treatment of solid organ cancers in individuals with A-T should avoid radiotherapy, as A-T confers exquisite hypersensitivity to ionising radiation, with increased susceptibility to second primary cancers and radiotherapy-related complications. Certain A-T families have also demonstrated adverse chemotherapy-related effects [122,123,124].

A-T is very rare, with a prevalence of 1 in 40,000 to 1 in 100,000, and the Hardy Weinberg principle dictates that this translates to a carrier frequency of at least 1 in 100. Monoallelic variants are associated with a moderately increased lifetime risk of breast cancer, over 20%, which is greatest for patients under 50 years of age [29,125,126]. Certain variants confer higher risk. In general, truncating variants appear to confer a higher risk than missense variants [32], but a recurrent missense variant c.7271T > G (p.Val2424Gly) is associated with a particularly high breast cancer risk, in the order of 60% [127]. In the UK, BRCA-equivalent screening for carriers of this particular high-risk missense variant is provided via the national very high-risk breast screening programme. Female heterozygous carriers of other pathogenic *ATM* variant are recommended to have increased breast surveillance starting from the age of 40. The frequency and duration of increased screening may be guided by the genotype and family history. MRI is favoured over mammography in homozygous/compound heterozygous carriers. Depending on the variant and the family history, prophylactic risk reducing surgery may be considered [50].

### 3.7. CHEK2

The checkpoint kinase protein *CHEK2* is a tumour suppressor involved in DNA damage response pathways. While the majority of pathogenic germline variants in *CHEK2* are associated with a moderately increased risk of breast cancer, certain variants may confer a higher risk of disease [128,129], and others with a more modest risk. The recurrent variant, c.1100delC, is prevalent in up to 2% of individuals of North European ancestry; while other truncating variants (c.444 + 1G > A, deletion of exons 9–10 (del5395)) are particularly common in individuals of Czech, Slovak or Polish ancestry, and are associated with at least a moderate disease risk. Carriers of high risk *CHEK2* variants are offered enhanced breast cancer surveillance [31,130,131], with the frequency and duration of enhanced screening guided by their family history. At present, in the UK, formal guidelines are lacking. It is accepted practice that the carriers of truncating variants in *CHEK2* are offered at least “moderate” risk screening, with annual mammograms starting from the age of 40 and continuing until the age of 50, with screening thereafter as part of the national breast cancer screening programme. The carriers of truncating variants in *CHEK2* that have a strong family history of breast cancer are offered high intensity surveillance, with annual mammograms between the ages of 40–60 years. In families where there is a strong family history of breast cancer, non-carriers of the familial *CHEK2* variant may still be offered moderate risk surveillance, because of the potential of other co-existing familial variants that have not been identified. Consideration of “very high risk” breast screening with annual MRI, as well as mammograms, should be considered for biallelic carriers of truncating *CHEK2* variants, but this is determined on a case-by-case basis. Missense variants in *CHEK2* are associated with a lower disease risk [32], and a recurrent missense variant (c.470T > C, p.I157T) is considered a low-penetrance allele [132]. Screening of the carriers of such variants is contingent on the family history rather than genotype alone.

Pathogenic variants in *CHEK2* are associated with bilateral disease and an oestrogen-positive phenotype, and in the affected AYA are associated with reduced disease-free rates and overall survival [133,134]. The risk conferred by the c.1100delC variant is greatly modified by family history and is also modified by co-inherited genetic modifiers. The use of a polygenic risk score, which is derived from the variation at 77 loci, can categorise the heterozygous carriers of CHEK2 c.1100delC into risk categories, with the OR of those in the highest quintile of PRS of 2.03 [0.86–4.78], compared to 0.52 [0.16–1.74] for those in the lowest quintile [135]. Because of the relatively common carrier frequency, homozygous carriers of the CHEK2 c.1100delC variant are not all that rare. Unlike other cancer predisposition genes, CHEK2 is not associated with a distinct recessive phenotype, but biallelic carriers have a higher cancer risk, and a tendency to develop cancer at younger ages [136,137].

### 3.8. BARD1

BRCA1 exists mostly as a stable heterodimer with BARD1 (BRCA1 associated RING domain 1). In this heterodimer form, the E3 ubiquitin ligase activity of BRCA1 is significantly increased [138]. This ligase activity is paramount in maintaining genomic integrity via the tumour suppressive function of BRCA1 [139]. The mechanisms by which this happens are not yet fully elucidated [27,140]. This ligase activity is disrupted when tumorigenic variants in *BRCA1* hinder the interaction between BARD1 and BRCA1, suggesting that similar complex-destabilising variants in *BARD1* may promote tumorigenesis. A recent multicenter association study of more than 113,000 women found that protein-truncating variants in *BARD1* were significantly associated with breast cancer, and most strongly with ER-negative and triple-negative disease [32]. This and other data suggest that *BARD1* variants confer a low-moderate risk of breast cancer susceptibility [32]. Association with other cancers, and optimal risk management, have yet to be determined.

## 4. Syndromic Causes of Early-Onset Breast Cancer

Highly penetrant pathogenic variants in *PTEN*, *TP53*, *STK11* and *CDH1* confer a lifetime risk of breast cancer greater than 40% [41,141,142]. Pathogenic variants in such genes are very rare in the general population, conferring a predisposition to specific cancer syndromes as detailed below.

### 4.1. PTEN

*PTEN* is well known as one of the most frequently somatically mutated tumour suppressors in human cancer. Somatic driver events in *PTEN* have been identified in an array of malignancies, including brain, prostate, and breast cancers [143]. Heterozygous germline variants give rise to a number of rare, autosomal dominant syndromes that are collectively described under the umbrella term of PTEN hamartoma tumour syndrome (PHTS), which encompasses Cowden syndrome [144], Bannayan-Riley-Ruvalcaba syndrome [145], and Proteus and Proteus-like syndrome [146]. *PTEN* is a multi-functional protein which exerts its tumour suppressor capabilities in numerous processes, including the maintenance of genomic stability, cell survival, proliferation, migration, invasion, and metabolism [147]. Germline pathogenic variants in *PTEN* predispose an individual to several types of benign and malignant neoplasia, conferring a high risk of benign breast disease and breast cancer [148]. The estimated lifetime breast cancer risk in an individual with PHTS is between 67–85% [141], similar to that conferred by germline variants in *BRCA1*/*BRCA2*. The tumour phenotype of PHTS also includes benign and malignant neoplasia of the thyroid, kidney, endometrium, skin and gastrointestinal tract [148]. The carriers of germline *PTEN* variants often present in childhood or adolescence with non-neoplastic features, including macrocephaly, developmental delay, or arteriovenous malformations [149].

In addition to screening for breast and other related malignancies, *PTEN* variant carriers may be offered surgical prophylaxis [50,150].

### 4.2. STK11

Heterozygous, pathogenic germline variants in the tumour suppressor gene *STK11* are associated with the autosomal dominant disorder Peutz-Jeghers syndrome (PJS). Affected patients develop benign intestinal hamartomas and are also at a highly increased risk of developing other malignancies [142,151], including of the pancreas [152], breast [153], and reproductive organs [154]. For female patients diagnosed with PJS, the risk for developing breast cancer rises to 8, 13, 31, and 45% at ages 40, 50, 60, and 70 years, respectively [142]. Current recommendations advise that annual breast screening with MRI begin as a young adult, around 25 years. Where there is a strong family history of breast cancer, a discussion of prophylactic surgery may be considered, although there is still insufficient evidence to support this action [153].

### 4.3. CDH1

Epithelial cadherin (E-cadherin) or cadherin-1 (CDH1) is a member of the cadherin superfamily. CDH1 is critical for cell adhesion, with roles in the regulation of cell polarity, differentiation and migration. E-cadherin expression is essential in normal embryonic development. Loss of E-cadherin is associated with the epithelial-mesenchymal transition (EMT), during which, epithelial cells demonstrate loss of apical-basal polarity and cellular adhesion, thereby acquiring the ability to migrate that leads, eventually, to metastatic dissemination. Loss of E-cadherin expression has been reported in gastric, colorectal, breast and ovarian cancers, with demonstrable changes in epithelial cell adhesion and motility. Loss of CDH1 activity results in increased cell motility leading to the increased metastatic ability of a tumour [155,156]. The associated cancer phenotype in heterozygous carriers of germline pathogenic variants in *CDH1* reflects a loss of function of E-cadherin, with increased risks of diffuse subtypes of gastric cancer, and/or lobular subtypes of breast cancer. The cumulative risk of lobular breast cancer in the female carriers of pathogenic *CDH1* variants by age 80 years is 39–55%, with the average age of diagnosis being 53 years, while the cumulative risk of diffuse gastric cancer in male and female carriers of such variants is approximately 33–42% [157], with a median age of onset of 38 and the youngest reported case aged only 14 at diagnosis.

In the carriers of a pathogenic *CDH1* variant with a personal or family history (first-/second-degree relative) of diffuse gastric cancer, the associated cancer predisposition syndrome is termed hereditary diffuse gastric cancer. At present, the only proven method to minimise the risk of diffuse gastric cancer in the carriers of such variants is prophylactic gastrectomy, and in classic HDGC families this is recommended between the ages of 20–30. However, pathogenic *CDH1* variants are increasingly being identified among patients with early-onset lobular breast cancer, in the absence of a personal or family history of diffuse gastric cancer [158,159,160], leading to some authors to propose revising the name of the associated syndrome to “hereditary diffuse gastric and lobular breast cancer”. Other authors propose that CDH1-associated hereditary lobular breast cancer may represent a distinct syndrome. However, in some patients, lobular breast cancer may predate a diagnosis of diffuse gastric cancer, and families with HLBC will be recategorized as having HDGC if a diagnosis of diffuse gastric cancer is made in the proband or a family member. The population frequency of hereditary diffuse gastric cancer is estimated to be approximately 5–10 per 100,000 [157]. However, among women diagnosed with bilateral lobular breast cancer prior to age 70, the yield of the detection of germline pathogenic *CDH1* variants is 7% [161]. Lobular phenotypes of breast cancer in the carriers of pathogenic germline CDH1 variants can be confirmed by demonstration of p120-catenin staining in the cytoplasm; with negative staining for β-catenin; but there are no histopathological/immunohistochemical tests that can differentiate between lobular cancer which is associated with germline CDH1 variants and that associated with somatic variants in this gene [157].

Prophylactic gastrectomy in carriers of *CDH1* pathogenic variants without a personal/family history of gastric cancer may not be appropriate, given the significant morbidity associated with this intervention. The optimum approach to manage a hypothetical risk of gastric cancer in such families has yet to be determined [162].

Breast screening with MRI from age 25–30 is recommended for the female carriers of pathogenic or likely pathogenic *CDH1* variants [50,163]. MRI is preferable to mammograms because of the lobular phenotype. Risk-reducing surgery may be considered in cases with a strong family history of breast cancer [164].

Appendiceal signet ring cancers, as well as colorectal cancers, have also been reported in the carriers of *CDH1* variants, who have non-malignant features including cleft lip and palate. Germline variants in *CDH1* have also been reported as a cause of non-syndromic cleft lip and palate, and of familial Blephorocheilodontic (BCD) Syndrome, with or without an associated cancer risk [165,166].

Therapeutic options in affected individuals, as well as the approach to surveillance in unaffected carriers, are also determined by their underlying genotype [40]. The knowledge that the carriers of such variants are at increased risk of radiation-induced second primary tumours means that radiotherapy is avoided where there is an alternative option that is at least non-inferior. In women affected by breast cancer, this means that mastectomy is favoured over breast-conserving surgery and radiotherapy. Similarly, surveillance protocols usually favour MRI compared to mammography—although the risk of mammography-induced cancer does not appear to be drastically increased in such individuals and may be considered as an option for those patients for whom MRI is not acceptable.

### 4.4. NF1

NF1 is a large protein with several domains. Germline pathogenic variants in *NF1* are associated with the autosomal dominant disorder, neurofibromatosis type 1 (NF1), which occurs as commonly as 1 in 2700 [167]. The features of NF1, which evolve over time, reflect an overgrowth of cells that are derived from differentiated glial cells, including melanocytes, Schwann cells, osteoblasts and chondrocytes. The most characteristic features of NF1 are the cutaneous manifestations, which include café au lait spots, axillary and inguinal freckling, and a tendency to develop simple and/or plexiform neurofibromas which give the condition its name. Affected individuals have an increased risk of developing an array of malignant tumours, including central nervous system glioma, gastro-intestinal stromal tumours, phaeochromocytomas, and pre-menopausal breast cancer [168]. The lifetime risk of breast cancer associated with pathogenic variants in *NF1* is approximately 20%, with a significant risk of developing breast cancer under the age of 50 [30,169], such that it has been suggested that patients with NF1 should be referred for enhanced breast cancer screening [170]. However, a recent study of more than 113,000 women was unable to definitively show an association between *NF1* and breast cancer risk [32]. Risk estimation and management should be individualised depending on the genotype and family history.

A clinical diagnosis of NF1 should be considered based on the clinical criteria as outlined in Table 1 [171]. Cutaneous findings may be subtle, and examination with a Woods lamp is indicated to identify features that might not be obvious otherwise. Germline mosaicism leading to segmental phenotypes is not uncommon. Confirmation by analysis of the NF1 gene facilitates cascade genetic testing, preimplantation testing and/or prenatal testing; and mRNA analysis is preferred to DNA sequencing to increase the diagnostic yield.

## 5. Putative High/Moderate Risk Genes

Variants in a number of other genes (including *MRE11*, *NBN*) have been postulated to be associated with an increased breast cancer risk, but their association has not, as of yet, been proven. Breast cancer has been reported as part of the phenotypic spectrum of the rare, recessive, NTHL1 tumour predisposition syndrome, with a median age of onset of 49 years (38–63) [172]. More common features of this condition include colorectal polyps (adenomatous, hyperplastic or sessile serrated), as well as colorectal and extracolonic cancers. Exploring the association between these candidate genes and breast cancer risk is beyond the scope of this review

## 6. Common, Low-Risk Variants

Hundreds of single nucleotide polymorphisms (SNPs) at loci scattered throughout the genome have been identified that are associated with a very slightly increased risk of breast cancer [173,174]. Most low-risk SNPs are located in intragenic regions.

While the individual risk conferred by these SNPs is small, they are at least additive; homozygous carriers are at an increased risk of disease over heterozygotes [174]. Low-penetrance SNPs are not routinely evaluated during the clinical assessment of breast cancer risk, though it is estimated that these SNPs may account for up to 18% of the inherited risk of breast cancer [173]. It is possible that the combined effect of co-inherited low-penetrance SNPs may be associated with a significantly increased risk of disease. Research is ongoing to determine if polygenic risk scores (PRS) can be utilised to stratify breast cancer risk in the general population and in high-risk variant carriers [175,176,177,178,179]. Most PRS have been derived from data related to individuals of European Caucasian ancestry, and therefore may not be applicable for other patient groups.

## 7. Overview of Environmental and Lifestyle Risk Factors for Breast Cancer

A number of environmental and lifestyle factors have been identified as risk factors for breast cancer. For breast cancer, these environmental and lifestyle factors include an exposure to ionizing radiation, exogenous hormones and reproductive choices, diet and alcohol consumption, obesity, and physical inactivity [180,181,182].

### 7.1. Hormonal Contraception and Reproductive Preferences

Between 2017–2019, 14% of women aged 15–49 years in the United States were using oral contraceptive pills (OCP) [183]. OCP use decreases with increasing age: approximately 1 in 5 women aged 15–19 and 20–29 use a form of OCP, versus 1 in 10 women aged 30–39 and 1 in 15 women aged 40–49 [183]. The risk of developing breast cancer as the result of OCP use has been a controversial debate for many years. Previous studies [184] showing an association to breast cancer have been criticised as outdated, given that today’s contraceptives offer lower levels of oestrogen, newer progestins, and newer delivery routes [185]. In 2017, using nationwide registries from 1995–2012, a large prospective cohort study of 1.8 million Danish women investigated the association between contemporary hormonal contraceptive (CHC) use and breast cancer [186]. When compared to women who had never used hormonal contraception, a relative risk of breast cancer of 1.2 was identified for women who were current or recent CHC users [186]. This risk increased from 1.09 for users of less than 1 year to 1.38 for users of more than 10 years [186]. For women taking CHC in excess of 5 years, the risk persisted following cessation of use. The relative risk for women using a progestin-only intrauterine system was also elevated at 1.21 versus never-users [186]. The overall absolute risk of breast cancer for all methods was modest and translated to approximately 1 extra breast cancer for every 7690 women using hormonal contraception for 1 year [186]. The absolute risk of invasive breast cancer was significantly more modest for those aged under 35 years, at approximately 1 extra case for every 50,000 women using CHC [186]. However, the context in which these findings are interpreted is important. CHC use is associated with a decreased risk of developing ovarian, endometrial, and colon cancers, and overall cancer risk may be lower in CHC users than in never-users [187,188].

In addition, the Danish study did not account for the confounding influence of factors such as alcohol consumption, physical inactivity, and breastfeeding. When considering the contraceptive and non-contraceptive benefits of CHC use, the overall benefit likely outweighs the small increased risk of breast cancer for most women. Patient factors such as a family history of disease, BMI, age, and intended duration of use should be considered when choosing a CHC method [189,190].

The age of someone’s first full-term pregnancy and their number of pregnancies can modulate breast cancer risk. The short-term risk of developing breast cancer increases for approximately 20 years, peaking after 5 years, following a pregnancy, and most significantly for those with a prior family history of disease [191,192]. This elevated hazard ratio is not observed for women whose first pregnancy occurred under the age of 25 years [191,192]. However, the overall lifetime risk of developing breast cancer is reduced for women whose first pregnancy was before the age of 30 [192]. A late first pregnancy (>35 years of age) is associated with an increased lifetime risk of breast cancer [192]. This further demonstrates that the risk factors of breast cancer differ between the AYAs and older adult population.

### 7.2. Obesity

Body mass index (BMI) is used as a tool for indicating nutritional status and is calculated by dividing a person’s weight in kilograms by the square of their height in metres (kg/m^2^). The World Health Organisation (WHO) defines pre-obesity (overweight) and obesity as a BMI ≥ 25 Kg/m^2^ and ≥30 Kg/m^2^, respectively [108]. The rates of obesity are rising similarly in Ireland, the UK, and the USA, where almost 70% of adults are overweight, and one in three is classed as obese [193,194,195]. Several studies have consistently demonstrated the association between increased BMI and an array of health conditions, including diabetes, hypertension, cardiovascular diseases [196], and cancers including postmenopausal breast cancer [197,198,199]. While the literature is somewhat conflicting, several studies indicate an inverse association between breast cancer risk and a greater BMI during adolescence and early adult years ([200,201,202] and references therein). This risk then inverts at a certain point in adulthood. A meta-analysis of over 1000 studies demonstrated a relative risk of 1.1 per 5 BMI units of postmenopausal breast cancer, particularly oestrogen receptor-positive disease, in women with a greater BMI [203]. Obesity at the time of diagnosis is associated with a poorer prognosis and 30% higher overall recurrence and mortality risk [204,205,206].

### 7.3. Physical Activity

Several studies have observed a favourable association between moderate physical activity and reduced breast cancer risk [207,208,209,210,211,212,213,214,215]. This reduction in cancer risk may be as significant as 20%, including for those with a family history of disease [214]. The benefit of low and high physical activity has been observed for both pre- and postmenopausal women [212], although it has been suggested that the most benefit may be derived from high levels of physical activity throughout adolescence and adulthood [210]. While breast cancer risk reduces with an increasing intensity of exercise [216], an optimal exercise regime to reduce cancer risk is yet to be determined. The World Cancer Research Fund recommends at least 30 min of moderate physical activity daily and an overall reduction in sedentary behaviours [217]. Considering the previously mentioned paradoxical effect of high BMI on pre- and postmenopausal breast cancer risk, the risk reduction conferred by physical activity is likely to be mediated through factors other than weight control. It has been suggested that physical activity may mediate breast cancer risk by decreasing insulin levels [209] and influencing insulin-like growth factors and binding proteins [218,219].

### 7.4. Diet

Studies into how diet can influence breast cancer risk are difficult to conduct given confounding factors such as alcohol consumption and the accuracy of nutrient measurement. Many of these studies to date have yielded conflicting and inconclusive results with regards to breast cancer risk. Some studies have indicated that a Mediterranean diet that is rich in fruits and vegetables, fish and olive oil, decreases the incidence of breast cancer [220], particularly oestrogen-receptor-negative disease [221,222,223]. A 2010 meta-analysis reported a diet that is rich in fruits and vegetables is associated with a reduced risk of breast cancer [224]. This relationship was not observed in a prospective study of over 993,000 women [225]. A study of nearly 91,000 women identified a 15% reduced risk of premenopausal breast cancer that was associated with a higher fruit and vegetable intake (2.9 servings/day) during adolescence and early adulthood [226].

Studies investigating the impact of dietary fat intake and breast cancer risk have also yielded conflicting results. A large US cohort study of 188,736 postmenopausal women found that dietary fat intake contributed directly to the risk of invasive postmenopausal breast cancer [227]. Women consuming the highest fat (40.1% energy from fat) had breast cancer rates 11–22% higher than those of women consuming the lowest amounts of fat (20.3% energy from fat). A subsequent meta-analysis of several cohort studies failed to replicate a significant association between dietary fat intake and breast cancer risk [228]. While inconclusively a risk factor for disease onset, several studies have found that a low-fat dietary intake is associated with a lower incidence of deaths following breast cancer diagnosis [225,229,230].

It had previously been postulated that as soy products contain phytoestrogens (isoflavones), excessive consumption could lead to an increase in oestrogen levels, and thus increase the risk of breast cancer [231]. However, nations with the highest soy consumption observe the lowest incidence and death rates of breast cancer. Several studies and meta-analyses have indicated that a moderate consumption of soy products confer a protective effect against pre- and postmenopausal breast cancer [232,233,234,235].

### 7.5. Alcohol Consumption

Alcohol consumption has been associated with several cancer types [236]. Alcohol consumption of any kind has been consistently associated with an increased risk of breast cancer [236,237,238,239,240,241,242]. As little as one drink per day, or 3–6 per week, contributes to this risk [237,238,240,243]. When compared to low-level drinkers (<60 drinks/year), those who had ever participated in binge drinking or who had blacked out showed increased risk of disease [243,244]. For each 10 g of alcohol consumed per day there was a 12% increase in breast cancer risk identified [238]. There were no differences observed for different alcohol types. Breast cancer risk increases linearly with cumulative lifetime alcohol consumption [243]. The same pattern of association and risk is observed for alcohol consumption levels between the ages of 18 and 40 years and for those over 40 years of age [243]. The proportion of breast cancers that can be attributed to alcohol varies from 2% in the USA [245] to 5% in Western Europe [246]. In countries where the overall alcohol consumption is higher or binge drinking is common, such as in Ireland and Italy, the proportion of breast cancers that can be attributed to alcohol is as high as 12% [246,247].

### 7.6. Previous Irradiation/Prior Childhood Cancers

It is well established that individuals who are exposed to ionising radiation during childhood, adolescence, or young adulthood are at an increased risk of developing a subsequent breast cancer. This radiation exposure may occur in diagnostic or therapeutic settings, such as in the case with Hodgkin lymphoma [248,249], or environmental, through the exposure to radiation via atomic bomb or nuclear disaster [250]. The risk of radiation-induced breast cancer is greatest for children who are exposed between the ages of 10 and 14 years, while exposure after 40 years of age increases the risk of breast cancer only marginally [251]. The risk of disease is also dependent on the dose of radiation given. This highlights the importance of the dosage during mammographic screening for breast cancer, given the low levels of radiation a patient is exposed to at regular intervals over a number of decades [252,253]. Several computational simulations of radiation-induced breast cancer in high-risk individuals suggest an optimal screening regime of MRI starting at age 25, with subsequent combined use of MRI with mammography starting at age 30 [254,255,256].

### 7.7. Breast Cancer Risks in Sexual and Gender Minorities (SGMs)

The risk of breast cancer in non-binary, transgender or intersex individuals is different to that of cisgender individuals, being significantly modified by factors such as hormone therapy, gender-confirming surgery, and surveillance, as well as those factors associated with breast cancer risk in cis-gender individuals. Breast cancer risk among transwomen has been found to be lower than that of ciswomen, but significantly increased when compared to cisgender males—in the order of 46-fold higher [257].

Identification of a high risk of breast cancer among such individuals is important, to direct screening and other risk-reducing strategies. Careful consideration should be given to such interventions, given the significant psychological distress that may be associated with them. An inclusive and sensitive approach to the risk estimation and management of individuals from sexual and gender minorities is critical [258].

Many transmen may choose to undergo bilateral mastectomy (“top surgery”) as part of their transition. Cases of breast cancer occurring in residual breast tissue in transmen who undergo such surgery have been reported [259,260,261,262,263]. The identification of a high risk of breast cancer in the individuals undergoing such surgery is important, so that an oncologic/preventative approach can be undertaken rather than the standard approach for masculinizing chest surgery. Depending on the health system, oncologic surgeries are likely to be prioritised over cosmetic surgeries, and therefore identification of a high breast cancer risk may determine waiting times for such surgery, and impact whether or not an insurance provider will cover the operation.

## 8. Holistic Risk Assessment

To provide an accurate estimate of risk in AYAs (and indeed in older patients), a holistic approach is required, bearing in mind lifestyle and reproductive factors as well as breast density, family history and heritable predisposition [264]. The risk factors outlined in this paper are summarised in Table 2.

Several models have been developed to assist clinicians in quantifying an individual’s lifetime risk of breast cancer, and since the discovery of the genes that are associated with monogenic predisposition, many have been developed to also include an estimation of the risk of carrying a high-risk variant in a breast cancer susceptibility gene [265].

### 8.1. Gail and Claus Models

The Breast Cancer Risk Assessment Tool, traditionally known as the Gail model, was developed in 1989 [266], and the Claus model since the early 90s [267].

The Claus model, which is based on data from the Cancer and Steroid Hormone study, allows an estimation of cancer risk that is based on family history and hereditary factors only [267]. The Gail model includes data regarding ethnicity, age, age at menarche, age at first pregnancy, family history, and the presence of atypical ductal hyperplasia. Initially developed using data from the Breast Cancer Detection Demonstration Project [266], which primarily included white women, the model has been adapted to include data related to black, Hispanic, Asian, and Pacific Islander women, using data from the Contraceptive and Reproductive Experiences (CARE) study, the California Surveillance, Epidemiology, and End Results (SEER) Program, the San Francisco Bay Area Breast Cancer Study, the California Cancer Registry, and the Asian American Breast Cancer Study [268,269,270]. The model underperforms for certain ethnic groups and is not accurate for patients with a previous history of invasive or in situ ductal or lobular breast carcinoma, nor for those with a preceding history of breast wall irradiation. Furthermore, it is not accurate for use in the known carriers of pathogenic variants in *BRCA1* or *BRCA2*, or other breast cancer susceptibility genes.

### 8.2. BRCAPRO

The BRCAPRO model is a Bayesian tool that incorporates information regarding population frequencies of pathogenic variants in *BRCA1* and *BRCA2*, penetrance estimates, as well as personal and family history of cancer—including data related to unaffected as well as affected relatives—to generate estimates of breast cancer risk as well as the probability of identifying a high-risk germline variant [271].

### 8.3. Tyrer-Cuzick Model

The Tyrer-Cuzick model includes data from the International Breast Intervention Study. Compared to other models, this tool allows the incorporation of genotypes related to other cancer susceptibility genes which include *BRCA1* and *BRCA2*, as well as family history, reproductive risk factors and ductal atypical hyperplasia [272].

### 8.4. BOADICEA and CanRisk

The Breast and Ovarian Analysis of Disease Incidence and Carrier Estimation Algorithm (BOADICEA) model incorporates information regarding *BRCA1* and *BRCA2* genotype, as well as polygenic risk and family history. BOADICEA has now been superseded by CanRisk [273]. CanRisk is a CE-marked tool which allows a clinician to enter data pertaining to lifestyle and reproductive risk factors, as well as family history and germline genetic risk factors—including high risk traits and polygenic risk scores. The tool allows an individualised estimation of lifetime breast cancer risk in the carriers and non-carriers of variants in *BRCA1*, *BRCA2*, *PALB2*, *CHEK2*, *ATM*, *RAD51C*, *RAD51D*, and *BRIP1*, and considers these modifiers of disease which may inform risk management. The tool does not permit genotype-specific information, so the user should bear this in mind when considering variants associated with reduced penetrance. The adage of “garbage in, garbage out” is particularly relevant when using these tools—care should be taken to verify family history and genotype where possible.

For individuals that have not yet had germline genetic testing, this tool can also estimate the a priori probability of identifying a pathogenic germline variant in one of these genes. In the UK, this is used to direct testing, as current guidelines permit NHS-funded germline testing where this probability is at least 10%—AYAs with breast cancer will meet this threshold with relatively little or no family history depending on the age at diagnosis.

The tool does not include information related to syndromic causes of breast cancer, precluding use of this tool to generate risk estimates in the known carriers of pathogenic variants in *CDH1*, *PTEN*, *TP53*, or *STK11.* In order to identify the carriers of such variants, the clinician should be alert to the phenotypes associated with rare syndromic causes of breast cancer.

Where PTEN hamartoma tumour syndrome is a consideration, the Cleveland Clinic PTEN calculator is a useful tool to estimate the likelihood of identifying a germline pathogenic variant in *PTEN* [274]. Clinical diagnostic criteria have been developed for this and other syndromic disorders, which may be useful in directing testing, or, importantly, in providing a clinical diagnosis to help guide management where germline genetic testing is uninformative [153,157,275,276].

### 8.5. Deep Learning and Emerging AI

Current screening for breast cancer relies on the physical exam, mammography, and, in selected patients, MRI. Mammography largely relies on the 5th edition of the BI-RADS scoring system to score images 0 to 6, with 0 being incomplete or inconclusive imaging and 6 being known, biopsy-proven malignancy.

Recently, advancements in artificial intelligence (AI) have led to the development of systems that are potentially capable of surpassing human experts in breast cancer prediction.

Zhu et al. analysed mammograms that were obtained in 6369 women without breast cancer, 1609 of whom developed screening-detected breast cancer, and 351 of whom developed interval invasive disease. This case–case–control study found that AI or deep learning systems outperformed clinical risk factors, including breast density, in detecting the screening-detected cancer risk but underperformed for detecting interval cancer risk [277].

Another recent article in the journal of Nature compared an AI system to human experts in the United Kingdom and USA in predicting breast cancer. To assess the AI’s performance in the clinical setting, the authors curated a large dataset from the UK and USA. They showed that the AI system resulted in an absolute reduction of 5.7% and 1.2% (USA and UK) in false positives and 9.4% and 2.7% in false negatives [2]. Furthermore, in an independent study of six radiologists, the AI system outperformed all human readers; the area under the receiver operating characteristic curve (AUC-ROC) for the AI system was greater than the AUC-ROC for the average radiologist by an absolute margin of 11.5% [278].

Ming et al. investigated the classification of lifetime breast cancer risk based on three different machine learning (ML) algorithms and the BOADICEA model in 112,587 individuals [279]. ML algorithms were found to have greater predictive accuracy than that of BOADICEA, reclassifying 35.3% of women in different risk categories [279]. The largest impact was observed in the screening for women younger than 50 years [279].

Further studies are required to explore the translational applications of these approaches.

## 9. Testing: When, Who, How and Why

In the UK and other countries with public health systems, germline genetic testing is often restricted to single genes or narrow gene panels and offered only to individuals who fulfill certain criteria. However, there is an increasing move towards broader gene panels, or whole exome/genome approaches. Broader testing optimises the cost- and time-efficiency and maximises the diagnostic yield. However, such testing also increases the potential for uncertainty and unexpected results. Some proponents of this approach argue that the identification of unexpected variants is an opportunistic advantage—facilitating risk-reducing strategies that may not otherwise have been offered based on the personal/family history. Broader approaches are particularly useful in individuals with atypical phenotypes, or where more than one heritable cancer predisposition syndrome is suspected. Finding more than one germline pathogenic variant, known as Multilocus Inherited Neoplasia Alleles Syndrome, is not all that rare; this is a particular risk in certain ethnic groups where the prevalence of founder mutations is high. “Double heterozygosity” or “transheterozygosity” of variants in both *BRCA1* and *BRCA2* is a rare event, and the associated phenotype is a mix of those found in *BRCA1* and *BRCA2* heterozygous carriers [275]. Double heterozygosity is a particular risk in individuals of Ashkenazi Jewish heritage, where carrier frequency of variants in *BRCA1* or *BRCA2* is relatively high.

Much of the additional diagnostic yield of broad cancer gene panels relates to the identification of variants associated with moderate, or uncertain, risks of breast cancer. This creates significant challenges in the clinical management of the carriers of such variants—as well as carrier and non-carrier relatives. In an AYA with a strong personal and family history of breast cancer, a variant associated with a moderate or low penetrance is likely to account only for a proportion of the disease risk. The residual risk may be attributable to environmental, lifestyle, or unidentified risk factors; however, a proportion may also be attributed to other co-inherited germline genetic variants that have not been detected. Risk management in such carriers should therefore consider family history and other modifying risk factors as well as the genotype; in non-carriers in families where a moderate risk allele has been identified, continued surveillance and/or other risk-reducing strategies may still be warranted.

## 10. Conclusions

Breast cancer in AYAs is relatively rare, and a significant proportion of risk is attributable to high-risk germline genetic variants, such that germline genetic testing should always be considered in affected individuals. A “negative” germline genetic test does not exclude the possibility of heritable risk factors, and enhanced cancer screening in the proband and close relatives is warranted and should be guided by the family history. For accurate risk estimation, a holistic approach is needed, that considers not only high-risk genotypes, but other modifiers of risk—including reproductive and lifestyle risk factors, and co-existing genetic modifiers, including the polygenic risk score, where available. Careful phenotyping in an AYA with breast cancer is crucial to avoid missing syndromic causes of breast cancer. In individuals for whom the lifetime breast cancer risk is estimated to be high, consideration should be given to surveillance, as well as chemo- and/or surgical prophylaxis. Furthermore, the opportunity to instigate changes to modify risk, such as lowering alcohol intake, reducing body weight, or adjusting contraception, should be explored, as summarised in Table 2.

## Figures and Tables

**Table 1:** Clinical diagnostic criteria for NF1.

Clinical Diagnostic Criteria for NF1. Requires at Least Two of the Following:
≥6 café au lait macules, bilaterally localised >5 mm diameter pre-puberty >15 mm post-puberty
≥2 neurofibromas of any type OR one plexiform Neurofibroma
Bilateral axillary/inguinal freckling
Optic pathway glioma
≥2 Lisch nodules (iris hamartomas) or two or more choroidal abnormalities
Osseous lesions Sphenoid Dysplasia Tibial pseudoarthrosis
Parent with NF1
Pathogenic ***NF1*** variant

**Table 2:** Summary of risk factors for breast cancer.

Risk Factor	Risk	Action	Population Frequency of Pathogenic Variants
**Inherited Predisposition**			
**Monogenic Variants**			
*TP53*	80–90%	Yearly breast MRI starting at 20 years	1/3555 to 1/5476
*BRCA1*	65–79%	MRI and/or mammogram starting at 25–30 years	1/381
*BRCA2*	61–77%	MRI and/or mammogram starting at 25–30 years	1/277
*PALB2*	44% to 63%	MRI and/or mammogram starting at 25–30 years	1/770
*RAD51C*	15–29%	Annual mammogram from 40–50/60 depending on family history	1/880
*RAD51D*	14–28%	Annual mammogram from 40–50/60 depending on family history	1/1680
*ATM*	OR 2.10 (1.71–2.57)	Increased screening with MRI starting at 40 years	1/100
*CHEK2*	OR 2.54 (2.21–2.91)	Annual mammography from 40–50 years, general screening thereafter	1/100
*BARD1*	OR 2.09 (1.35–3.23)	Risk management not yet determined	1/1100
**Syndromic causes**			
*PTEN*	~85%	MRI and/or mammogram starting at 25–30 years	1/200,000
*STK11*	45–54%	MRI starting at 25 years	1/25,000–1/280,000
*CDH1*	23–68%	MRI starting at 25–30 years	<0.1/100,000
*NF1*	SMR 5.20 (2.38–9.88)	Risk management not yet determined	1/1900–1/3000
**Common, low-risk variants**	*Low*	No action	
**Lifestyle factors**			
**Hormonal contraception and reproductive preferences**			
**CHC use**	*Modest*	CHC use should be directed by family history. In general, the benefits largely outweigh increased risk of breast cancer	
**Age at first pregnancy**	Decreased risk if first pregnancy <30 years Increased risk if first pregnancy >35 years		
**Obesity**	Decreased risk with greater BMI in childhood and adolescents Increased risk with greater BMI in adulthood		
**Physical Activity**	Reduced risk associated with moderate physical activity. Further reduction with increasing activity level	Optimal exercise regime yet to be determined. WHO recommends ≥30 min of moderate physical activity daily, and an overall reduction in sedentary behaviours	
**Diet**	Inconclusive data		
**Alcohol Consumption**	Increased risk with any consumption, increasing linearly with cumulative lifetime alcohol consumption		
**Radiation Exposure/Childhood Cancer**	Greatest risk for children exposed between the ages of 10 and 14 years	Risk management not yet determined. Computational algorithms suggest MRI starting at age 25, combined with mammography starting at age 30	
**Sexual and Gender Minorities**	Risk for transgender women lower than cisgender women but significantly higher than cisgender men	Identification of other high-risk factors to direct screening and other risk-reducing strategies	

CHC: contemporary hormonal contraceptive; BMI: body mass index; WHO: World Health Organisation.
